# Editorial: Education in oral health

**DOI:** 10.3389/froh.2023.1315663

**Published:** 2023-11-08

**Authors:** Fawad Javed, Ricardo D. Coletta

**Affiliations:** ^1^ Department of Orthodontics and Dentofacial Orthopedics, Eastman Institute for Oral Health, University of Rochester, Rochester, NY, United States; ^2^ Department of Oral Diagnosis and Graduate Program in Oral Biology, Piracicaba Dental School, University of Campinas, Piracicaba, Brazil

**Keywords:** education, oral health, community-based education, oral diseases, quality of life

**Editorial on the Research Topic** Education in oral health

Oral diseases (OD), such as dental caries, periodontal disease, and premalignant and malignant lesions, affect millions of individuals worldwide. The consequences of such disorders extend beyond the mouth, impacting systemic health, nutrition, speech, and social well-being (1). In addition, oral health issues that remain undiagnosed and/or untreated in a timely manner can result in significant healthcare expenditures and a compromised quality of life (QoL) in many instances (Barranca-Enríquez and Romo-González, 2). The solution to this challenge begins with comprehensive education in oral health (EIOH), a critical foundation that empowers individuals to make informed decisions about their oral care. It empowers individuals with the knowledge and skills needed to maintain good oral hygiene practices, make healthy dietary choices, and seek timely dental/medical care. Education also dispels myths and misconceptions, reducing dental anxiety and fostering positive attitudes toward oral health. Moreover, EIOH is not limited to the public; it is equally crucial for healthcare professionals (3–5). In other words, it is empirically sound to assert that EIOH functions as a pivotal cornerstone in the prevention of OD and the promotion of holistic health.

Nearly a year ago, *Frontiers in Oral Health* launched a Research Topic titled “Education in Oral Health.” This Research Topic focused on exploring strategies for educating both patients and healthcare providers in effectively managing OD, including those referenced earlier. Furthermore, it also emphasized on educational components aimed at enhancing community awareness regarding risk factors, prevention, and early detection of OD. The primary objective of this editorial is to furnish an overview and summarize the principal findings of studies (Barranca-Enríquez and Romo-González, Balkaran et al., Lawal et al., Chen et al.) that were published within the scope of the Research Topic titled “Education in Oral Health” after a vigilant peer-review process. Of the four studies (Barranca-Enríquez and Romo-González, Balkaran et al., Lawal et al., Chen et al.), two were original (Balkaran et al., Lawal et al.) and the others were a comprehensive review (Barranca-Enríquez and Romo-González) and a systematic reviews (Chen et al.).

## Objectives and primary outcomes of original studies

The study by Balkaran et al. was performed in Trinidad and Tobago, a twin island country situated off the northern edge of the South American mainland. This study (Balkaran et al.) aimed to assess the efficacy of a dental workshop tailored for dental practitioners, allied dental health professionals, and students, with a focus on individuals with special needs, within this region. According to the methodology, a feedback study was undertaken between 2019 and 2020, comprising two surveys: the initial survey was administered during the workshop, while the subsequent survey was conducted one year later. In the follow-up phase, an online survey tool was employed to evaluate the workshop, inviting participants to provide comments and suggestions. Most of the attendees were females (∼82%) and dental students and dentists comprised of 50.3% and 38.9% of the attendees, respectively. At least 90% of the attendees gave positive feedback for the workshop; and 80% reported that the workshop helped increase their knowledge (Balkaran et al.). The study concluded that there is a notable need for dental services tailored to individuals with special needs, along with an ongoing need for educational opportunities. Furthermore, the findings suggested that such workshops can have a demonstrable impact on the quality of patient care (Balkaran et al.).

In a cross-sectional questionnaire-based survey, Lawal et al. assessed the influence of oral hygiene habits on the QoL of school-going adolescents residing in Ibadan, Nigeria. In this study, individuals aged 15.1 ± 1.16 years were included (Lawal et al.). Approximately 60% of the respondents reported that they brushed teeth twice daily and nearly 68% spend at least three-minutes in toothbrushing. Adolescents who brushed their teeth at least twice and performed flossing of interproximal spaces had significantly better oral-health related QoL scores compared with adolescents that performed toothbrushing once daily and did not perform interdental flossing (Lawal et al.). This study (Lawal et al.) concluded that toothbrushing at least twice daily and interproximal flossing at least once a day positively impacts the QoL of individuals (Barranca-Enríquez and Romo-González, Balkaran et al., Lawal et al., Chen et al.).

## Objectives and primary outcomes of the comprehensive review

The comprehensive review by Barranca-Enríquez and Romo-González evaluated the significance of oral health and its relationship with overall well-being. In this regard, the authors searched indexed databases for pertinent randomized controlled trials (RCTs) as well as review articles (Barranca-Enríquez and Romo-González). The authors reported that oral health is a multifaceted concept encompassing various dimensions, including physical, psychological, social, and environmental aspects, all of which are integral to comprehensive health and overall well-being. Similarly, the oral cavity holds significance as the psychological epicenter of primary physiological needs and emotional satisfaction (Barranca-Enríquez and Romo-González). Consequently, the mouth assumes a pivotal role in fostering a sense of interconnectedness and contributing to the formation of one's self-identity. The study (Barranca-Enríquez and Romo-González) emphasized on the need for community-based strategies for oral health promotion as well as disease prevention.

## Objectives and primary outcomes of the systematic review

Chen et al. systematically reviewed studies that addressed the prevention and management of dental caries (DC) among patients with type 2 diabetes mellitus (T2DM). The authors explored several indexed databases including PubMed/Medline, EMBASE and ISI web of knowledge to identify the pertinent literature. The initial search yielded 909 studies out of which two RCTs were included and processed for data extraction. This systematic review (Chen et al.) provides evidence that consistent and comprehensive EIOH, involving the utilization of interdental cleaning aids, mouthwash, moisturizing gel, as well as salivary substitutes are effective in managing oral inflammation and contributing to the control of DC in individuals with T2DM.

## Summary of published studies

In summary, the published studies (Barranca-Enríquez and Romo-González, Balkaran et al., Lawal et al., Chen et al.) provide region-specific insights into dental care and oral hygiene practices, with a focus on special needs and adolescents, respectively. These studies (Barranca-Enríquez and Romo-González, Balkaran et al., Lawal et al., Chen et al.) contribute to our understanding of the importance of tailored dental services, education, and oral hygiene habits in improving oral health and overall quality of life within these specific populations and geographic contexts.

## Recommendations/perspectives

Early EIOH should be integrated into school curricula from a young age. By teaching children about the importance of oral hygiene, the role of nutrition, and the consequences of neglecting oral health, we can instill lifelong habits that prevent dental issues. Likewise, community-based initiatives can play a role in educating masses particularly underserved populations about oral health and its relationship with well-being in general. Mobile dental clinics, school-based programs, and outreach campaigns can make a substantial impact by reaching individuals who may lack access to regular dental care. Dentists, dental hygienists, nurses, and physicians should receive ongoing training in the latest advancements in oral healthcare. This may help ensure that they provide evidence-based care and promote preventive measures effectively.
